# Association of maternal pre-existing atherosclerotic cardiovascular disease and neonatal and long-term offspring outcomes: a nationwide mother–child paired cohort

**DOI:** 10.1093/hropen/hoaf074

**Published:** 2025-11-25

**Authors:** Danbee Kang, Jihye Heo, Ki Hong Choi, Taegyun Park, Ji-Hee Sung, Taek Kyu Park, Joo Myung Lee, Juhee Cho, Jeong Hoon Yang, Young Bin Song, Joo-Yong Hahn, Seung-Hyuk Choi, Hyeon-Cheol Gwon, Soo-Young Oh

**Affiliations:** Center for Clinical Epidemiology, Samsung Medical Center, Sungkyunkwan University School of Medicine, Seoul, Republic of Korea; Department of Clinical Research Design and Evaluation, SAIHST, Sungkyunkwan University, Seoul, Republic of Korea; Center for Clinical Epidemiology, Samsung Medical Center, Sungkyunkwan University School of Medicine, Seoul, Republic of Korea; Department of Clinical Research Design and Evaluation, SAIHST, Sungkyunkwan University, Seoul, Republic of Korea; Division of Cardiology, Department of Internal Medicine, Heart Vascular Stroke Institute, Samsung Medical Center, Sungkyunkwan University School of Medicine, Seoul, Republic of Korea; Department of Clinical Research Design and Evaluation, SAIHST, Sungkyunkwan University, Seoul, Republic of Korea; National Health Insurance Service, Wonju, Republic of Korea; Department of Obstetrics and Gynecology, Samsung Medical Center, Sungkyunkwan University School of Medicine, Seoul, Republic of Korea; Division of Cardiology, Department of Internal Medicine, Heart Vascular Stroke Institute, Samsung Medical Center, Sungkyunkwan University School of Medicine, Seoul, Republic of Korea; Division of Cardiology, Department of Internal Medicine, Heart Vascular Stroke Institute, Samsung Medical Center, Sungkyunkwan University School of Medicine, Seoul, Republic of Korea; Center for Clinical Epidemiology, Samsung Medical Center, Sungkyunkwan University School of Medicine, Seoul, Republic of Korea; Department of Clinical Research Design and Evaluation, SAIHST, Sungkyunkwan University, Seoul, Republic of Korea; Division of Cardiology, Department of Internal Medicine, Heart Vascular Stroke Institute, Samsung Medical Center, Sungkyunkwan University School of Medicine, Seoul, Republic of Korea; Division of Cardiology, Department of Internal Medicine, Heart Vascular Stroke Institute, Samsung Medical Center, Sungkyunkwan University School of Medicine, Seoul, Republic of Korea; Division of Cardiology, Department of Internal Medicine, Heart Vascular Stroke Institute, Samsung Medical Center, Sungkyunkwan University School of Medicine, Seoul, Republic of Korea; Division of Cardiology, Department of Internal Medicine, Heart Vascular Stroke Institute, Samsung Medical Center, Sungkyunkwan University School of Medicine, Seoul, Republic of Korea; Division of Cardiology, Department of Internal Medicine, Heart Vascular Stroke Institute, Samsung Medical Center, Sungkyunkwan University School of Medicine, Seoul, Republic of Korea; Department of Obstetrics and Gynecology, Samsung Medical Center, Sungkyunkwan University School of Medicine, Seoul, Republic of Korea

**Keywords:** atherosclerotic cardiovascular disease, neonatal outcome, offspring outcome, adverse pregnancy outcome, mother–child paired cohort

## Abstract

**STUDY QUESTION:**

Is maternal pre-existing atherosclerotic cardiovascular disease (ASCVD) associated with neonatal and long-term offspring outcomes?

**SUMMARY ANSWER:**

Maternal pre-existing ASCVD is independently associated with consistent increases in neonatal and early childhood adverse outcomes.

**WHAT IS KNOWN ALREADY:**

Maternal cardiovascular health may influence offspring development through intrauterine and early-life mechanisms. However, the impact of maternal pre-existing ASCVD on neonatal and long-term offspring’s neurodevelopmental outcomes remains poorly understood.

**STUDY DESIGN, SIZE, DURATION:**

We conducted a nationwide cohort study linking maternal and child health data from the Korean National Health Insurance Service between 2005 and 2019.

**PARTICIPANTS/MATERIALS, SETTING, METHODS:**

Pre-existing ASCVD was defined by diagnostic codes for myocardial infarction, ischemic stroke, or angina prior to conception. Offspring outcomes included neonatal complications (congenital malformations, sepsis, neonatal intensive care unit admission) and neurodevelopmental disorders (developmental delay, seizure, or attention-deficit hyperactivity disorder), with follow-up through 2020. Among 5 461 222 live births (3 640 815 unique mothers), 145 315 (2.7%) were delivered by women with pre-existing ASCVD before pregnancy. Propensity score matching (1:4) was used to adjust for baseline maternal characteristics.

**MAIN RESULTS AND THE ROLE OF CHANCE:**

Offspring of mothers with ASCVD had increased risk of congenital malformations (adjusted odds ratio [aOR] 1.09, 95% CI: 1.07–1.12), neonatal intensive care unit admission (aOR 1.19, 95% CI: 1.16–1.22), and neonatal sepsis (aOR 1.11, 95% CI: 1.07–1.15). The risk of neurodevelopmental disorders was also elevated (adjusted hazard ratio 1.08, 95% CI: 1.07–1.10). The absolute risk differences were consistent regardless of the presence of adverse pregnancy outcomes during pregnancy.

**LIMITATIONS, REASON FOR CAUTION:**

As with all observational studies, residual confounding could not be ruled out. Only live births are included.

**WIDER IMPLICATIONS OF THE FINDINGS:**

Maternal pre-existing ASCVD was associated with modest but consistent increases in neonatal and early childhood adverse outcomes. These associations appeared to persist regardless of the presence of adverse pregnancy outcomes, suggesting a possible independent contribution of maternal ASCVD on offspring development. Our findings suggest the importance of identifying and monitoring pregnancies of women with cardiovascular disease and indicate that early neurodevelopmental surveillance may be considered in affected children.

**STUDY FUNDING/COMPETING INTEREST(S):**

This study was supported by the Patient-Centered Clinical Research Coordinating Center (PACEN) funded by the Ministry of Health & Welfare, Republic of Korea (grant number: HC21C0123) and supported by the Bio&Medical Technology Development Program of the National Research Foundation (NRF) funded by the Korean government (MSIT) (No. RS-2024-00440881). No competing interests are reported.

**TRIAL REGISTRATION NUMBER:**

ClinicalTrials.gov, NCT06406998.

WHAT DOES THIS MEAN FOR PATIENTS? Some women have heart and blood vessel problems, like heart attacks or strokes, before they become pregnant. This study looked at whether these health problems in mothers might affect the health of their babies. We found that children born to mothers with these conditions had slightly higher chances of certain health concerns at birth and were also somewhat more likely to need follow-up for developmental health as they grew. These patterns were observed even when there were no major pregnancy complications. These findings suggest that careful monitoring during pregnancy and early support after birth may be helpful for both mothers and their children. More research is needed to better understand these connections and how to support healthy outcomes for families.

## Introduction

Although the majority of atherosclerotic cardiovascular disease (ASCVD) occurs in men, ASCVD is also the leading cause of death even in women worldwide (Tsao *et al.*, 2022). In particular, both the prevalence of myocardial infarction (MI) and its mortality have been rising in younger and middle-aged women, while a decrease in cardiovascular mortality across the general population has been observed over the past decades (Arora *et al.*, 2019). As ASCVD becomes more prevalent among women of reproductive age, concerns have emerged regarding its potential impact on their offspring (Roos-Hesselink *et al.*, 2019).

Emerging evidence suggests that maternal cardiovascular health may influence fetal development and long-term child outcomes through pathways involving placental insufficiency, inflammation, and altered fetal programming (de Souza Lima *et al.*, 2024). Previous studies have shown associations between maternal risk factors, such as hypertension and diabetes, and adverse neonatal outcomes (Li *et al.*, 2020; Wang *et al.*, 2023). Based on these findings, we hypothesized that maternal pre-existing ASCVD may adversely influence both neonatal and long-term neurodevelopmental outcomes in offspring.

However, little is known about the effects of maternal ASCVD on offspring outcomes, particularly beyond the neonatal period. Most existing research has focused on short-term obstetric complications or maternal outcomes, with few studies examining long-term developmental risks in children born to mothers with ASCVD (Honigberg *et al.*, 2019; Li *et al.*, 2025). Moreover, prior studies have often been limited by small sample sizes and have lacked linkage between maternal cardiovascular history and child health data (Lameijer *et al.*, 2015, 2019). To address this gap, we used a nationwide population-based mother–child cohort to investigate the association between maternal pre-existing ASCVD and adverse neonatal, as well as long-term neurodevelopmental, outcomes in offspring.

## Materials and methods

### Study design

We conducted a nationwide retrospective cohort study using the Korean National Health Insurance Service (K-NHIS) database, which represents the entire population of Korea and contains national records of all covered inpatient and outpatient visits, procedures, and prescriptions from 2004 to 2020 (Kim *et al.*, 2021). In addition, the K-NHIS claims database includes data from national health screening examinations conducted as part of a standardized health screening program provided to all insured persons every 2 years (Cheol Seong *et al.*, 2017). Mother–child linkage was performed using unique resident registration numbers in the K-NHIS database, which enables near-complete matching between maternal and offspring records. However, because the linkage is possible only for registered live births, our cohort included all live births from 1 January 2005 to 31 December 2019 with a washout period of 2004 and a follow-up period of 31 December 2020. K-NHIS linked all claim data of mothers to all claim data of offspring (N = 5 461 222).

### Study participants

The study cohort included 3 640 815 unique mothers, corresponding to a mean parity of 1.5 births per woman. Women with pre-existent ASCVD were defined as those who presented to the clinic with angina pectoris, MI, or ischemic stroke before the estimated last menstrual period. The last menstrual period date was estimated by using an algorithm previously validated in administrative healthcare databases to estimate gestational age (Margulis *et al.*, 2013). Using the database, we selected offspring born to women with pre-existent ASCVD (N = 145 315). Because ASCVD exposure was defined prior to each pregnancy, only births that occurred after the diagnosis of ASCVD were included in the exposed group, meaning that each woman contributed only one pregnancy to the ASCVD cohort. Then, we selected 1:4 matched offspring born to women without pre-existent ASCVD using propensity score (PS) matching (N = 581 260) based on maternal and perinatal characteristics (see details in the Statistical analysis section). The final sample size was 726 575 (Supplementary Fig. S1). The need for informed consent was waived, as this study was conducted using anonymized claims data. This study was approved by the Institutional Review Board of Samsung Medical Center, Republic of Korea (No. 2021-08-107).

### Measurements

#### Definitions of outcomes

K-NHIS claims for inpatient and outpatient visits, procedures, and prescriptions are coded using the International Classification of Diseases, 10th revision (ICD-10) (Chun *et al.*, 2009). Maternal adverse pregnancy outcomes (APOs) included preterm delivery, pre-eclampsia or eclampsia, other hypertensive disorders of pregnancy, small-for-gestational-age, and gestational diabetes. Neonatal outcomes of offspring included chromosomal abnormalities, major congenital malformations, and neonatal composite morbidities. Chromosomal abnormalities were identified based on diagnostic codes ICD-10: Q90–Q99 in the claims database. Major congenital malformations were also identified by diagnostic records based on ICD-10 codes defined by the European Surveillance of Congenital Anomalies classification (EUROCAT Central Registry, 2013). Neonatal composite morbidities were defined as the composite of morbidities, which included any of the following: transient tachypnea, respiratory distress syndrome, necrotizing enterocolitis, intraventricular hemorrhage, bronchopulmonary dysplasia, and sepsis. Admission to the intensive care unit and occurrence of seizure events were considered adverse infant outcomes. Vital status was obtained from death certification collected by Statistics Korea at the Ministry of Strategy and Finance of Korea (Lee *et al.*, 2017). Long-term offspring outcomes were also defined based on prespecified neurological and neurodevelopmental diagnoses codes: autism spectrum disorder, attention-deficit hyperactivity disorder, cerebral palsy, any developmental delay including motor or cognitive delay, epileptic and febrile seizures and tics, and stereotypic behavior. In a validation study, positive predictive values were high (range: 82–98%) (Straub *et al.*, 2021). Registration of these diagnoses is required for reimbursement, ensuring near-complete ascertainment. A full list of ICD-10 codes used for outcome definitions is provided in Supplementary Table S1.

#### Confounders

We considered a broad range of covariates as potential confounders or proxies of potential confounders: maternal age at delivery, income, residential area at delivery, history of stillbirth, and maternal comorbidities including hypertension, diabetes, hyperlipidemia, and malignancy. Data on age and income at the time of the first screening exam were obtained from the insurance eligibility database. Income level was categorized by percentile groups (medical aid or ≤25th, >25th–≤50th, >50th–≤75th, and >75th percentile). Residential area at the time of the first screening exam was classified as metropolitan or rural. Metropolitan areas were defined as Seoul, six metropolitan cities, and 15 cities with a population >500 000 officially designated as municipal cities. Maternal comorbidities within the year prior to delivery defined the presence of disease codes in the claims database. Additionally, national health screening examinations collected information on behaviors such as smoking, drinking, and physical activity for mothers through standard questionnaires, as well as anthropometric measurements, including body mass index. None of the variables included in the PS had missing data because they were derived from mandatory administrative claim records. Variables from the national health screening program, such as smoking status and body mass index, were available only for a subset of women who underwent health examinations. Thus, a sensitivity analysis restricted to women who underwent health examinations (N = 313 024) was conducted to confirm that repeated pregnancies did not affect the associations.

### Statistical analysis

For 1:4 matching, we generated a PS for women with pre-existing ASCVD using birth date, income, residential area, maternal age at delivery, number of birth prior current births, maternal history of stillbirth, and comorbidities before pregnancy (hypertension, diabetes mellitus, hyperlipidemia, and malignancy). PS matching was performed using a greedy algorithm (caliper = 0.1). Differences in baseline covariables between the two groups were evaluated before and after PS matching using the absolute standardized mean difference with a value of >0.1 indicating a significant difference (Austin, 2011). As a sensitivity analysis, we also analyzed the full population, adjusting for the same covariates to assess the robustness of the results.

Absolute risk differences (RDs) and 95% CIs were calculated using the Wald normal approximation with binomial variance, equivalent to the delta method for independent proportions. For short-term outcomes, including major congenital malformations, neonatal composite morbidities, seizures, intensive care unit (ICU) admission, and neonatal death, odds ratios (ORs) with 95% CIs were estimated for women with pre-existing ASCVD compared with those without ASCVD. For long-term outcomes, including neurodevelopmental disorders and death, all live births were followed from birth until the occurrence of the outcome, death, or administrative censoring on 31 December 2020. Cox proportional hazards models were used to estimate hazard ratios (HRs) with 95% CIs for long-term outcomes. Because the study was based on nationwide insurance claim data, individuals were followed continuously unless death occurred. For neurodevelopmental outcomes, death before diagnosis was considered a competing event; therefore, cumulative incidence was estimated using Fine–Gray subdistribution hazard models, which accounted for death as a competing risk.

As a sensitivity analysis, we evaluated whether the associations between maternal ASCVD and offspring outcomes persisted among women with and without APOs, to assess whether ASCVD had an impact independent of APO-mediated pathways.

Because ASCVD status was defined prior to each pregnancy, only births that occurred after the diagnosis of ASCVD were included in the exposed group, ensuring that each woman contributed only one pregnancy to the ASCVD cohort. In contrast, women without ASCVD could have multiple pregnancies during the study period. Therefore, to account for within-mother correlation among repeated births in the non-ASCVD group, we used mixed-effects models in all analyses. Additionally, a sensitivity analysis restricted to first-time mothers (N = 411 159) was conducted to confirm that repeated pregnancies did not affect the associations. All analyses were two-sided, and *P*-values <0.05 were considered statistically significant. Statistical analyses were performed using SAS version 9.2 (SAS Institute, Inc, Cary, NC, USA).

## Results

**Figure 1.**
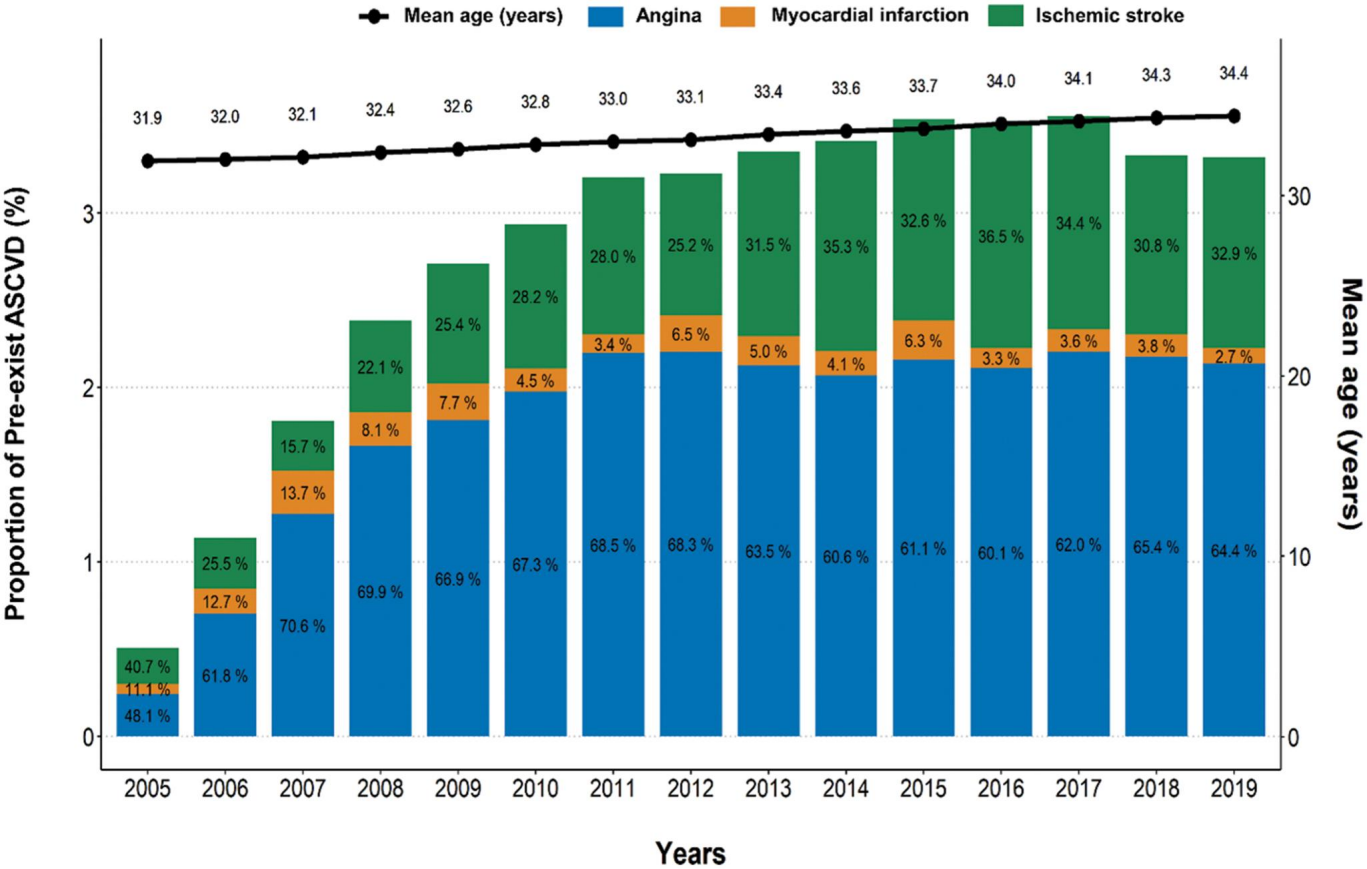
**Trends in maternal age and pre-existing atherosclerotic cardiovascular diseases among new mothers**. Bar graph indicates the overall prevalence of pre-existing ASCVD among new mothers. Black line indicates mean maternal age. ASCVD, atherosclerotic cardiovascular diseases.

Among the 5 461 222 live births (3 640 815 unique mothers), 145 315 (2.7%) were born to women with pre-existing ASCVD before pregnancy. The prevalence of pre-existing ASCVD tended to increase over the study period along with the steady increase in maternal age at childbirth (Fig. 1). The median duration from diagnosis of ASCVD to pregnancy was 3.9 years (IQR: 1.8–6.9 years). Women with pre-existing ASCVD were more likely to be older, live in rural areas, and have more cardiovascular comorbidities than those without pre-existing ASCVD (Supplementary Table S2). After PS matching, all standardized mean differences of baseline characteristics between groups were <0.1 (Table 1). The mean number of prior births was 1.49 in women without ASCVD and 1.51 in those with ASCVD (SMD = 0.032). The proportion of singleton pregnancies was also similar (56.8% vs 55.6%; SMD = 0.009).

**Table 1.** Baseline characteristics of the matched cohort according to the presence or absence of maternal pre-existing atherosclerotic cardiovascular diseases.

	Without pre-existing ASCVD (n = 581 260)	Pre-existing ASCVD (n = 145 315)	SMD
**Maternal age, years**	33.31 (3.94)	33.27 (4.17)	0.010
<35 years	362 990 (62.5)	90 264 (62.1)	0.007
≥35 years	218 270 (37.6)	55 051 (37.9)	
**Income level**			
Medical aid or ≤25th	19 823 (3.4)	6171 (4.2)	0.029
>25th to ≤50th	106 662 (18.4)	27 373 (18.8)	0.013
>50th to ≤75th	282 461 (48.6)	69 496 (47.8)	0.015
>75th percentile	172 314 (29.6)	42 275 (29.1)	0.012
**Residence area, rural**	334 586 (57.6)	77 819 (53.6)	0.061
**Body mass index**			
Underweight (<18.5 kg/m^2^)	38 289 (6.6)	8832 (6.1)	0.021
Normal (18.5 to <23 kg/m^2^)	159 815 (27.5)	37 997 (26.2)	0.030
Overweight (23 to <25 kg/m^2^)	29 004 (5.0)	7460 (5.1)	0.007
Obese (≥25 kg/m^2^)	24 817 (4.3)	6814 (4.7)	0.020
Unknown	329 335 (56.7)	84 212 (58.0)	0.026
**Smoking status**			
Never	231 030 (39.8)	55 265 (38.0)	0.035
Ever	21 443 (3.7)	5951 (4.1)	0.021
Unknown	328 787 (56.6)	84 099 (57.9)	0.026
**History of stillbirth**	4025 (0.7)	1721 (1.2)	0.051
**Comorbidities before pregnancy**			
Hypertension	5595 (1.0)	2636 (1.8)	0.073
Diabetes mellitus	17 963 (3.1)	5894 (4.1)	0.052
Hyperlipidemia	22 960 (4.0)	7883 (5.4)	0.070
Malignancy	6992 (1.2)	2032 (1.4)	0.017
ASCVD	0 (0)	145 315 (100)	
Angina pectoris	0 (0)	72 245 (49.7)	
Myocardial infarction	0 (0)	10 843 (7.5)	
Ischemic stroke	0 (0)	59 634 (41.0)	
**Medication before pregnancy**			
Aspirin	5965 (1.0)	145 225 (99.9)	4.764
Statin	22 960 (4.0)	79 520 (54.7)	0.475
**Period of delivery**			
2005–2009	136 288 (23.4)	33 620 (23.1)	0.007
2010–2014	239 306 (41.2)	61 529 (42.3)	0.024
2015–2019	205 666 (35.4)	50 166 (34.5)	0.018
**Birth events**			
Cesarean delivery	246 674 (42.4)	67 496 (46.4)	0.081
Multiple gestation	20 196 (3.5)	6022 (4.1)	0.035
Birth weight, kg (N = 686 004)	3.18 (0.4)	3.16 (0.50)	0.045
**Previous number of births**	1.49 (0.62)	1.51 (0.62)	0.032
Singleton	330 366 (56.8)	80 793 (55.6)	0.009

Values are expressed as means (SDs) or n (%).

ASCVD, atherosclerotic cardiovascular diseases; SMD, standardized mean difference.

The risks of APOs including preterm birth, pre-eclampsia or eclampsia, other hypertensive disorders of pregnancy, and gestational diabetes were significantly higher in women with pre-existing ASCVD than in those without pre-existing ASCVD (Supplementary Table S3).

With regard to neonatal outcomes of offspring, no differences were observed in neonatal death (0.2% vs 0.2%, OR: 1.09, 95% CI: 0.94–1.25) and chromosomal abnormalities (0.1% vs 0.1%, OR: 0.99, 95% CI: 0.80–1.23) according to the presence or absence of pre-existing ASCVD. However, major congenital malformations (3.9% vs 4.2%, OR: 1.09, 95% CI: 1.06–1.12) and neonatal adverse events (5.1% vs 5.6%, OR: 1.12, 95% CI: 1.09–1.15) occurred more frequently in the offspring of women with pre-existing ASCVD than in the offspring of women without it. Among major congenital malformations, heart defects (2.4% vs 2.7%, OR: 1.10, 95% CI: 1.06–1.14) and digestive defects (0.3% vs 0.3%, OR: 1.17, 95% CI: 1.05–1.30) were notably more prevalent in offspring of women with pre-existing ASCVD. Among neonatal composite morbidities, neonatal sepsis (2.9% vs 3.2%, OR: 1.11, 95% CI: 1.07–1.15), respiratory distress syndrome (1.2% vs 1.5%, OR: 1.22, 95% CI: 1.16–1.28), and bronchopulmonary dysplasia (0.1% vs 0.1%, OR: 1.36, 95% CI: 1.14–1.62) were higher in the offspring of women with pre-existing ASCVD (Table 2).

**Table 2.** Neonatal adverse events of offspring according to the presence or absence of maternal pre-existing atherosclerotic cardiovascular diseases.

	Number of events (%)		Risk difference (95% CI)	Odds ratio (95% CI)
	Without pre-existing ASCVD (n = 581 260)	Pre-existing ASCVD (n = 145 315)		
**Major congenital malformations**	22 735 (3.9)	6163 (4.2)	0.33 (0.21–0.44)	1.09 (1.06–1.12)*
Nervous system	1417 (0.2)	373 (0.3)	0.01 (−0.02 to 0.04)	1.05 (0.94–1.18)
Eyes	350 (0.1)	87 (0.1)	0.00 (−0.01 to 0.01)	0.99 (0.79–1.26)
Ear, face, and neck	210 (0)	58 (0)	0.00 (−0.01 to 0.02)	1.11 (0.83–1.48)
Heart defects	14 054 (2.4)	3861 (2.7)	0.24 (0.15–0.33)	1.10 (1.06–1.14)*
Respiratory system	264 (0.1)	71 (0.1)	0.00 (−0.01 to 0.02)	1.08 (0.83–1.40)
Oral clefts	709 (0.1)	183 (0.1)	0.00 (−0.02 to 0.02)	1.03 (0.88–1.22)
Digestive system	1541 (0.3)	449 (0.3)	0.04 (0.01–0.08)	1.17 (1.05–1.30)*
Abdominal wall defects	222 (0)	61 (0)	0.00 (−0.01 to 0.02)	1.10 (0.83–1.46)
Urinary system	2887 (0.5)	754 (0.5)	0.02 (−0.02 to 0.06)	1.05 (0.96–1.13)
Genital system	542 (0.1)	142 (0.1)	0.00 (−0.01 to 0.02)	1.05 (0.87–1.26)
Limb	853 (0.2)	240 (0.2)	0.02 (−0.00 to 0.04)	1.13 (0.98–1.30)
Other malformations	823 (0.1)	194 (0.1)	−0.01 (−0.03 to 0.01)	0.94 (0.81–1.10)
**Neonatal composite morbidities** ^†^	29 351 (5.1)	8174 (5.6)	0.58 (0.44–0.71)	1.12 (1.09–1.15)*
Sepsis	16 988 (2.9)	4692 (3.2)	0.31 (0.21–0.41)	1.11 (1.07–1.15)*
Transient tachypnea	6283 (1.1)	1626 (1.1)	0.04 (−0.02 to 0.10)	1.04 (0.98–1.09)
Respiratory distress syndrome	7163 (1.2)	2176 (1.5)	0.27 (0.20–0.33)	1.22 (1.16–1.28)*
Necrotizing enterocolitis	142 (0)	31 (0)	0.00 (−0.01 to 0.01)	0.87 (0.59–1.29)
Intraventricular hemorrhage	179 (0)	41 (0)	0.00 (−0.01 to 0.01)	0.92 (0.65–1.29)
Bronchopulmonary dysplasia	487 (0.1)	165 (0.1)	0.03 (0.01–0.05)	1.36 (1.14–1.62)*
**Seizures**	12 975 (2.2)	3571 (2.5)	0.23 (0.14–0.31)	1.10 (1.06–1.15)*
**ICU admission**	30 606 (5.3)	8991 (6.2)	0.92 (0.79–1.06)	1.19 (1.16–1.22)*
**Neonatal death**				
Within 1 month	234 (0.04)	58 (0.04)	−0.00 (−0.00 to 0.00)	0.99 (0.74–1.32)
Within 1 year	894 (0.2)	243 (0.2)	0.01 (−0.01 to 0.04)	1.09 (0.94–1.25)

† Neonatal composite morbidities were defined as the composite of neonatal sepsis, transient tachypnea, respiratory distress syndrome, necrotizing enterocolitis, intraventricular hemorrhage, and bronchopulmonary dysplasia.

Variables included in propensity score matching: birth date, income, residential area, maternal age at delivery, number of birth prior current births, maternal history of stillbirth, and comorbidities before pregnancy (hypertension, diabetes mellitus, hyperlipidemia, and malignancy).

Statistical significance was assessed using logistic regression.

* Asterisks indicate statistical significance at *P* < 0.05.

ASCVD, atherosclerotic cardiovascular diseases; ICU, intensive care unit.

With regard to infant outcomes, ICU admission (5.3% vs 6.2%, OR: 1.19, 95% CI: 1.16–1.22) and seizure (2.2% vs 2.5%, OR: 1.10, 95% CI: 1.06–1.15) incidence were higher in offspring of women with pre-existing ASCVD than in offspring of women without it. In the full cohort (N = 5 461 222), the results were consistent with those of the matched analysis (Supplementary Table S4). Furthermore, analyses restricted to first-time mothers (N = 411 159; Supplementary Table S5) and to women who had participated in the national health screening program (N = 313 024; Supplementary Table S6) yielded findings that were consistent with the main results.

During the follow-up period (median, 7.8 years; IQR, 4.82–10.79 years), there was no significant difference in the risk of any cause of death between the two groups (Table 3). In contrast, the risk of neurodevelopmental disorders during follow-up was higher in offspring of women with pre-existing ASCVD than in those without ASCVD (21.1% vs 19.5%; HR: 1.08, 95% CI: 1.07–1.10; Table 3). The increased risk of neurodevelopmental disorders remained consistent in the competing risk analysis (Supplementary Table S7).

**Table 3.** Long-term developmental outcome in children born to mothers with atherosclerotic cardiovascular diseases.

	Number of events (Cumulative incidence %)		Risk difference (95% CI)	Hazard ratio (95% CI)
	Without pre-existing ASCVD (n = 581 260)	Pre-existing ASCVD (n = 145 315)		
**Neurodevelopmental disorders**	79 249 (19.5)	21 395 (21.1)	1.60 (1.37–1.83)	1.08 (1.07–1.10)*
Autism	3839 (0.8)	982 (0.9)	0.10 (0.05–0.15)	1.02 (0.95–1.09)
ADHD	13 370 (3.0)	3733 (3.3)	0.30 (0.20–0.40)	1.11 (1.07–1.15)*
Cerebral palsy	1711 (0.4)	496 (0.4)	0.00 (−0.04 to 0.04)	1.16 (1.05–1.28)*
Developmental delay	22 310 (5.0)	6070 (5.4)	0.40 (0.27–0.53)	1.08 (1.05–1.12)*
Motor developmental delay	11 299 (2.5)	3183 (2.8)	0.30 (0.21–0.39)	1.12 (1.08–1.17)*
Cognitive developmental delay	11 821 (2.6)	3116 (2.8)	0.20 (0.11–0.29)	1.05 (1.01–1.09)*
Epileptic and febrile seizures	49 126 (11.7)	13 205 (12.6)	0.90 (0.47–0.80)	1.08 (1.06–1.10)*
Tics and stereotypic behaviors	6725 (1.5)	1818 (1.6)	0.10 (0.03–0.17)	1.08 (1.02–1.13)*
**Any cause of death**	1396 (0.3)	359 (0.3)	0.00 (−0.03 to 0.03)	1.03 (0.92–1.15)

Variables included in propensity score matching: birth date, income, residential area, maternal age at delivery, number of birth prior current births, maternal history of stillbirth, and comorbidities before pregnancy (hypertension, diabetes mellitus, hyperlipidemia, and malignancy).

Statistical significance was assessed using Cox regression.

* Asterisks indicate statistical significance at *P* < 0.05.

ADHD, attention-deficit hyperactivity disorders; ASCVD, atherosclerotic cardiovascular diseases.

In stratified analyses by the presence or absence of APOs, the associations between maternal ASCVD and offspring outcomes remained consistent across subgroups (Table 4). For congenital malformations, the risk was slightly higher in offspring of mothers with ASCVD both without APOs (3.2% vs 3.4%; RD: 0.18%, 95% CI: 0.06–0.30; OR: 1.06, 95% CI: 1.02–1.10) and with APOs (5.9% vs 6.5%; RD: 0.60%, 95% CI: 0.33–0.87; OR: 1.11, 95% CI: 1.06–1.16). Similarly, neurodevelopmental disorders were more frequent in offspring of mothers with ASCVD in both subgroups, without APOs (18.7% vs 20.2%; RD: 0.99%, 95% CI: 0.76–1.23; HR: 1.08, 95% CI: 1.06–1.10) and with APOs (21.7% vs 23.9%; RD: 1.30%, 95% CI: 0.90–1.71; HR: 1.09, 95% CI: 1.06–1.13).

**Table 4.** Offspring adverse events according to the presence or absence of maternal pre-existing atherosclerotic cardiovascular diseases.

Neonatal/infant outcomes	Number of events (%)		Risk difference (95% CI)	Odds ratio (95% CI)
	Without pre-existing ASCVD	Pre-existing ASCVD		
**Without APOs (N = 537 557)**	N = 431 493	N = 106 064		
Chromosomal abnormalities	222 (0.1)	54 (0.1)	0.00 (−0.02 to 0.01)	0.99 (0.74–1.33)
Major congenital malformations	13 901 (3.2)	3611 (3.4)	0.18 (0.06–0.30)	1.06 (1.02–1.10)*
Neonatal composite morbidities^†^	16 949 (3.9)	4457 (4.2)	0.27 (0.14–0.41)	1.07 (1.04–1.11)*
ICU admission	11 024 (2.6)	3020 (2.9)	0.29 (0.18–0.40)	1.12 (1.07–1.16)*
Seizures	9202 (2.1)	2501 (2.4)	0.23 (0.12–0.33)	1.11 (1.06–1.16)*
**Neonatal death**				
Within 1 month	0	0	–	–
Within 1 year	34 (0.0)	5 (0.0)	0.00 (−0.01 to 0.00)	0.60 (0.23, 1.53)
**With APOs (N = 189 018)**	N = 149 767	N = 39 251		
Chromosomal abnormalities	202 (0.1)	51 (0.1)	0.00 (−0.05 to 0.04)	0.96 (0.71–1.31)
Major congenital malformations	8834 (5.9)	2552 (6.5)	0.60 (0.33–0.87)	1.11 (1.06–1.16)*
Neonatal composite morbidities^†^	12 402 (8.3)	3717 (9.5)	1.19 (0.87–1.51)	1.16 (1.12–1.20)*
ICU admission	19 582 (13.1)	5971 (15.2)	2.14 (1.74–2.53)	1.19 (1.16–1.23)*
Seizures	3773 (2.5)	1070 (2.7)	0.12 (−0.06 to 0.30)	1.08 (1.01–1.16)*
**Neonatal death**				
Within 1 month	234 (0.2)	58 (0.2)	−0.00 (−0.00 to 0.00)	0.95 (0.71–1.26)
Within 1 year	860 (0.6)	238 (0.6)	0.03 (−0.05 to 0.12)	1.06 (0.92–1.22)

Long-term outcome	Number of events (Cumulative incidence %)		Risk difference (95% CI)	Hazard ratio (95% CI)
	Without pre-existing ASCVD	Pre-existing ASCVD		
**Without APOs (N = 537 557)**	N = 431 493	N = 106 064		
Any neurodevelopmental disorders	57 676 (18.7)	15 229 (20.2)	0.15 (1.23–1.77)	1.08 (1.06–1.10)*
Any cause of death	267 (0.1)	59 (0.1)	−0.00 (−0.02 to 0.02)	0.89 (0.67–1.19)
**With APOs (N = 189 018)**	N = 149 767	N = 39 251		
Any neurodevelopmental disorders	21 573 (21.7)	6166 (23.9)	2.20 (1.73–2.67)	1.09 (1.06–1.13)*
Any cause of death	1129 (1.0)	300 (1.0)	0.00 (−0.11 to 0.11)	1.01 (0.89–1.15)

† Neonatal composite morbidities were defined as the composite of neonatal sepsis, transient tachypnea, respiratory distress syndrome, necrotizing enterocolitis, intraventricular hemorrhage, and bronchopulmonary dysplasia.

Variables included in propensity score matching: birth date, income, residential area, maternal age at delivery, number of birth prior current births, maternal history of stillbirth, and comorbidities before pregnancy (hypertension, diabetes mellitus, hyperlipidemia, and malignancy).

Statistical significance was assessed using logistic regression for Neonatal/infant outcomes and Cox regression for long-term outcome.

* Asterisks indicate statistical significance at *P* < 0.05.

APOs, adverse pregnancy outcomes; ASCVD, atherosclerotic cardiovascular diseases; ICU, intensive care unit.

## Discussion

In this nationwide cohort study of mother–child pairs, we found that 4.0 of women had pre-existing ASCVD. After generating a 1:4 PS matched cohort, offspring of women with pre-existing ASCVD were associated with slightly higher risks of major congenital malformations, neonatal and infant adverse events as well as long-term neurodevelopmental disorders.

In this study, we found that the prevalence of pre-existing ASCVD showed an increasing trend over the course of 10 years. Among ASCVD, angina was the most prevalent followed by ischemic stroke. Aging of the maternal population is a significant factor. National statistics indicate that the average age of first-time mothers has been rising steadily, correlating with increased risks of chronic health conditions, including ASCVD. Age-related physiological changes such as reduced vascular compliance and increased arterial stiffness may exacerbate the risk of developing cardiovascular conditions. Furthermore, there is an increasing prevalence of cardiovascular risk factors among younger populations, including obesity and hypertension (Driscoll and Gregory, 2020; Lihme *et al.*, 2024). Given the rising prevalence of pre-existing cardiovascular diseases and demographic shifts in maternal age, addressing pregnancy and childbirth in this population is essential, necessitating integrated and proactive healthcare strategies. Nevertheless, the modified World Health Organization Classification of Maternal Cardiovascular Risk includes arrhythmias, cardiomyopathy, or congenital heart disease, while ASCVD as a diagnosis is not included (Regitz-Zagrosek *et al.*, 2011). Also, the Cardiac Disease in Pregnancy (CARPREG) II score, which incorporates coronary artery disease as a lesion-specific predictor for cardiac complications during pregnancy, includes only cardiomyopathy-related disease (Silversides *et al.*, 2018), Given that the rate of ASCVD has been increasing over time, it is reasonable to consider women with pre-existent ASCVD as having a modified World Health Organization pregnancy classification of II–III.

The current study findings also highlight that the risks associated with maternal pre-existing ASCVD can potentially lead to long-term issues for the offspring. In this study, offspring born to women with pre-existing ASCVD were at higher risk of major congenital malformations, neonatal adverse events, and infant adverse events. The risk of neurodevelopmental disorders was also higher in offspring of women with pre-existing ASCVD than offspring of women without ASCVD. Although the relative increases in risk observed in this study were modest, the growing prevalence of ASCVD and its major risk factors may amplify the absolute number of affected pregnancies and offspring at a population level. From a public health perspective, even small relative effects may translate into meaningful increases in overall disease burden. Our stratified analyses showed that the associations between maternal ASCVD and adverse offspring outcomes were consistent irrespective of the presence of APOs. Although the effect estimates appeared slightly stronger among women with APOs, the overlapping CIs suggest partial rather than complete mediation. This pattern supports a potential direct contribution of maternal ASCVD to fetal development through vascular and inflammatory mechanisms, in addition to an indirect contribution mediated by APOs. Although previous Swedish and British Columbia cohort studies included a broad range of maternal cardiovascular conditions with relatively few women affected by ASCVD, pre-pregnancy maternal cardiovascular disease was associated with increased risks of neurodevelopmental disorders in offspring, consistent with our findings (Hossin *et al.*, 2024). In the Swedish nationwide cohort, 22 745 offspring of mothers with cardiovascular disease had a 1.17-fold (95% CI: 1.10–1.25) higher adjusted HR for ADHD. Similarly, in the British Columbia cohort, 22 010 offspring of mothers with cardiovascular disease showed a 1.14-fold (95% CI: 1.09–1.20) increased risk of ADHD.

These findings are also consistent with our original hypothesis that maternal cardiovascular health influences fetal development through pathways involving endothelial dysfunction, systemic inflammation, oxidative stress, and metabolic dysregulation, which may impair uteroplacental blood flow and fetal oxygen or nutrient supply (Palinski, 2014). These disturbances may negatively impact structural brain development in the offspring. Existing evidence suggests a link between pre-existing cardio-cerebral dysfunction and compromised placental circulation, which in turn can contribute to pregnancy-related and neonatal complications (Siegmund *et al.*, 2019). Medication exposure during pregnancy may also play a role, as fetal neurodevelopment is particularly susceptible to pharmacologic effects. A recent nationwide cohort study reported that first-trimester exposure to statins was not associated with congenital malformations overall, but high-intensity statin use showed increased risk (Kay *et al.*, 2025). Moreover, while observational data suggest statin use may be safe with respect to congenital anomalies, caution is still warranted given potential low birth weight or preterm effects (Chang *et al.*, 2021). Taken together, these vascular, inflammatory, and pharmacologic pathways may underlie the observed associations and warrant further investigation in future longitudinal and interventional studies. Our findings underscore the lack of evidence regarding the safety and efficacy of cardiovascular medications in pregnant women with ASCVD. Because pregnant and lactating women are routinely excluded from interventional studies, clinicians face uncertainty in balancing maternal cardiovascular benefits with potential fetal risks. There is a pressing need for ethically designed studies and systematic data collection to inform evidence-based clinical decisions in this population (Riley, 2024).

### Limitations

This study had several limitations. First, we utilized retrospective data. While our data were comprehensive, the retrospective nature of the data could have introduced biases including information bias, as we relied on previously recorded administrative data not originally intended for research purposes. However, such data are considered reliable and have been used in numerous peer-reviewed publications, as the K-NHIS routinely audits claims (Shin *et al.*, 2016; Lee *et al.*, 2017). Second, because deaths occurring before official registration are not captured in the national insurance database, our study cohort included registered live births only. Although we evaluated mortality within 1 month and 1 year after birth among offspring, selection bias may still exist, as severe malformations or pregnancy terminations resulting in fetal loss before registration could not be identified. Our findings suggest associations but cannot definitively conclude that pre-existing ASCVD caused the observed complications in pregnancy and the post-partum period. Third, the identification of ASCVD relied on hospital-reported ICD-10 codes; detailed clinical records were unavailable to verify the diagnosis. Therefore, there is the possibility that the prevalence of pre-existing ASCVD in pregnant women might have been overestimated. Fourth, we did not have detailed information about the treatment of ASCVD during pregnancy, such as specific medications, dosages, or adherence to treatment plans. In addition, data on assisted reproductive technology were limited because insurance coverage for assisted reproductive technology was introduced only recently in Korea, and detailed information on assisted reproductive technology procedures was not captured during the study period. Finally, this study was confined to Korea, underscoring the necessity for future replication across different nations, particularly in regions with diverse racial demographics.

## Conclusions

In summary, offspring of women with pre-existing ASCVD showed modest increases in the risk of major congenital malformations and in the frequency of neonatal or infant adverse events and long-term neurodevelopmental disorders. Given that an increasing number of women with pre-existing ASCVD are anticipated to become pregnant in the future, careful monitoring and appropriate support for reproductive-aged women with an underlying ASCVD remain important. Our findings underscore the need for further large-scale studies in diverse populations to confirm these associations and to elucidate the underlying biological mechanisms.

## Supplementary Material

hoaf074_Supplementary_Data

## Data Availability

We used the claim data provided by the Korean National Health Insurance Service (K-NHIS) database. Data can only be accessed by visiting the K-NHIS datacenter, after approval from the data access committee of K-NHIS. Those who want to access the data set of this study should contact the first author (dbee.kang@skku.edu), who will help with the process of contacting the K-NHIS.
