# Supplementary material for: Association of maternal pre-existing atherosclerotic cardiovascular disease and neonatal and long-term offspring outcomes: a nationwide mother–child paired cohort
Source: Hum Reprod Open. 2025 Nov 25;2025(4):hoaf074. doi: 10.1093/hropen/hoaf074 (PMC12690663; doi:10.1093/hropen/hoaf074)
Supplement: hoaf074_Supplementary_Data [file hoaf074_supplementary_data.docx]

**Supplementary Data**

**Association of Maternal Atherosclerotic Cardiovascular Disease with Neonatal and Long-Term Offspring Outcomes: A Nationwide Mother-Child Paired Cohort**

Danbee Kang, Jihye Heo1, Ki Hong Choi, Taegyun Park, Ji-Hee Sung, Taek Kyu Park, Joo Myung Lee, Juhee Cho, Jeong Hoon Yang, Young Bin Song, Joo-Yong Hahn, Seung-Hyuk Choi, Hyeon-Cheol Gwon, Soo-Young Oh

**Table of Contents**

**Supplementary Table S1.** Definitions of study outcomes

**Supplementary Table S2.** Baseline characteristics of study participants before propensity-score matching

**Supplementary Table S3.** Adverse pregnancy outcomes according to the presence or absence of maternal pre-existing ASCVD

**Supplementary Table S4.** Neonatal adverse events of offspring according to the presence or absence of maternal pre-existing ASCVD in full cohort (N= 5461222)

**Supplementary Table S5.** Sensitivity analysis for Neonatal adverse events of offspring according to the presence or absence of maternal pre-existing ASCVD among only first-time mothers (N=411159)

**Supplementary Table S6.** Sensitivity analysis for Neonatal adverse events of offspring according to the presence or absence of maternal pre-existing ASCVD among only participated in health examination (N=313024)

**Supplementary Table S7.** Competing risk analysis of neurodevelopmental disorders in offspring by maternal pre-existing ASCVD status

**Supplementary Figure S1.** Study flowchart

**Supplementary Table S1. Definitions of study outcomes**

|  | **Codes** |
| --- | --- |
| **Delivery** |  |
| Delivery | R3131–R3148, R4351–R4362, R4380, R4507-–R4520, R5001-–R5002, RA311-–RA318, RA361-–RA362, and RA380-–RA434 |
| **Adverse pregnancy outcomes** |  |
| Preterm delivery | O42, O601, O603, P073, and P590 |
| Pre-eclampsia or eclampsia | O11, O14, or O15 |
| Small-for-gestational-age | O365 or birth weight<2.5kg |
| Other hypertensive disorders of pregnancy | O10, O13, or O16 |
| Gestational diabetes | O244 |
| **Offspring outcomes** |  |
| **Major congenital malformations** |  |
| Nervous system | Q00-–Q07 |
| Eyes | Q100, Q104, Q106-–Q109, Q11-–Q12, Q130-–Q134, Q136-–Q139, and Q14-–Q15 |
| Ears, face, and neck | Q16, Q176-–Q178, Q183, and Q188 |
| Heart defects | Defects of cardiac chambers and connections (Q20); cardiac septal defects (Q21); pulmonary and tricuspid valve defects (Q22); aortic and mitral valve defects (Q23); other heart defects (Q24); defects of the great arteries (Q25); and defects of the great veins (Q260, Q262-–Q269) |
| Respiratory system | Q300, Q321-–Q329, Q330, Q332-–Q335, Q337-–Q339, and Q34 |
| Oral clefts | Q35-–Q37 |
| Digestive system | Tongue, mouth, and pharynx (Q380, Q383-–Q389); esophagus (Q39); upper alimentary tract (Q402-–Q409); small intestine (Q41); large intestine (Q42); other malformations of the intestine (Q431-–Q439); gallbladder, bile ducts and liver (Q44); other malformations of the digestive system (Q45); and diaphragmatic hernia (Q790) |
| Abdominal wall defects | Q792-–Q793 and Q795 |
| Urinary system | Q60, Q611-–Q619, Q620-–Q626, Q628-–Q629, Q630-–Q632, Q634-–Q639, Q64, and Q794 |
| Genital organs | Q50-–Q51, Q520-–Q522, Q524, Q526, Q528-–Q529, and Q54-–Q56 |
| Limb | Q660-–Q661, Q679, Q681-–Q682, Q686-–Q689, and Q70-–Q74 |
| Other malformations | Q750, Q77, Q782-–Q788, Q80-–Q81, Q820-–Q824, Q826-–Q829, Q860, Q890, and Q893-–Q894 |
| **Neonatal composite morbidities** |  |
| Sepsis | R65, A40, A41, O753 |
| Transient tachypnea | P221 |
| Respiratory distress syndrome | P220, P228, P229 |
| Necrotizing enterocolitis | P77 |
| Intraventricular hemorrhage | P520, P521, P522 |
| Bronchopulmonary dysplasia | P271 |
| **Infant outcomes** |  |
| Intensive care unit admission | AJ111, AJ211, AJ311, AJ121, AJ221, AJ321, AJ101, AJ201, AJ301, AJ131, AJ231, AJ331, AJ141, AJ241, AJ341, AJ144, AJ244, AJ301, AJ251, AJ351, AJ161, AJ161, AJ261 |
| Seizures | G40, G41, R56, G253 |
| **Long-term outcomes** |  |
| **Neurodevelopmental disorders** |  |
| Attention deficit hyperactivity disorder | F88, F89, F840, F841, F844, F845, F848, F849, F900, F901, F902, F908, F909 |
| Autism | F84 |
| Cerebral palsy | G80 |
| Developmental delay | F82, R26, R27, F70, F71, F72, F73, F78, F79, F83, F800, F801, F802, F808, F809, F810, F811, F812, F813, F818, F819, R480, R488 |
| Motor developmental delay | F82, R26, R27 |
| Cognitive developmental delay | F70, F71, F72, F73, F78, F79, F83, F800, F801, F802, F808, F809, F810, F811, F812, F813, F818, F819, R480, R488 |
| Tics and stereotypic behavior | R25, F950, F951, F952, F958, F959, F984, F985 |
| Epileptic and febrile seizures | G40, G41, R56, G253 |

**Supplementary Table S2. Baseline characteristics of study participants before propensity-score matching**

|  | **Without pre-existing ASCVD (N=5315907)** | **Pre-existing ASCVD (N=145315)** | **SMD** |
| --- | --- | --- | --- |
| **Maternal age, years** | 32.46 (4.18) | 33.27 (4.17) | 0.194 |
| <35 years | 3705772 (69.7) | 90264 (62.1)) | 0.161 |
| ≥35 years | 1610135 (30.3) | 55051 (37.9) |  |
| **Income level** |  |  |  |
| Medical aid or ≤25^th^ | 203895 (3.9) | 6171 (4.2) | 0.008 |
| >25^th^–≤50^th^ | 1048665 (19.7) | 27373 (18.8) | 0.023 |
| >50^th^–≤75^th^ | 2606737 (49.0) | 69496 (47.8) | 0.024 |
| >75^th^ percentile | 1456610 (27.4) | 42275 (29.1) | 0.038 |
| **Residence area, rural** | 1720204 (32.4) | 77819 (53.6) | 0.438 |
| **Body mass index** |  |  |  |
| Underweight (<18.5 kg/m^2^) | 313588 (5.9) | 8832 (6.1) | 0.008 |
| Normal (18.5-<23 kg/m^2^) | 1300520 (24.5) | 37997 (26.2) | 0.039 |
| Overweight (23-<25 kg/m^2^) | 238416 (4.5) | 7460 (5.1) | 0.030 |
| Obese (≥25 kg/m^2^) | 204063 (3.8) | 6814 (4.7) | 0.042 |
| Unknown | 3259320 (61.3) | 84212 (58.0) | 0.069 |
| **Smoking status** |  |  |  |
| Never | 1888312 (35.5) | 55265 (38.0) | 0.052 |
| Ever | 172323 (3.2) | 5951 (4.1) | 0.045 |
| Unknown | 3255272 (61.2) | 84099 (57.9) | 0.069 |
| **History of stillbirth** | 32337 (0.6) | 1721 (1.2) | 0.061 |
| **Comorbidities before pregnancy** |  |  |  |
| Hypertension | 487126 (0.9) | 2636 (1.8) | 0.327 |
| Diabetes mellitus | 144878 (2.7) | 5894 (4.1) | 0.074 |
| Hyperlipidemia | 178741 (3.4) | 7883 (5.4) | 0.101 |
| Malignancy | 55634 (1.0) | 2032 (1.4) | 0.032 |
| ASCVD | 0 (0) | 145315 (100) |  |
| Angina pectoris | 0 (0) | 72245 (49.7) |  |
| Myocardial infarction | 0 (0) | 10843 (7.5) |  |
| Ischemic stroke | 0 (0) | 62227 (42.8) |  |
| **Medication before pregnancy** |  |  |  |
| Aspirin | 47386 (0.9) | 145225 (99.9) | 12.849 |
| Statin | 178968 (3.4) | 79520 (54.7) | 0.543 |
| **Period of delivery** |  |  |  |
| 2005-2009 | 1957965 (36.9) | 33620 (23.1) | 0.302 |
| 2010-2014 | 1910643 (35.9) | 61529 (42.3) | 0.131 |
| 2015-2019 | 1447569 (27.2) | 50166 (34.5) | 0.158 |
| **Birth events** |  |  |  |
| Cesarean delivery | 2175398 (40.9) | 67496 (46.4) | 0.112 |
| Multiple gestations | 163514 (3.1) | 6022 (4.1) | 0.057 |
| Birth weight, kg (N = 4,952,591) | 3.18 (0.48) | 3.16 (0.50) | 0.052 |
| **Previous number of births** | 1.38 (0.59) | 1.51 (0.62) | 0.220 |
| Singleton | 3538168 (66.6) | 80793 (55.6) | 0.440 |

Values are expressed as means ± standard deviations or N (%).

Abbreviations: ASCVD, atherosclerotic cardiovascular disease; SMD, standardized mean difference; PS, propensity score

**Supplementary Table S3. Adverse pregnancy outcomes according to the presence or absence of maternal pre-existing atherosclerotic cardiovascular disease**

|  | **Number of events (%)** | | **Odds ratio**  **(95% CI)** |
| --- | --- | --- | --- |
|  | **Without pre-existing ASCVD** | **Pre-existing ASCVD** |  |
| **Preterm delivery** | 28,376 (4.9) | 8,760 (6.0) | 1.25 (1.22, 1.28) |
| **Pre-eclampsia or eclampsia** | 8,016 (1.4) | 2,560 (1.8) | 1.28 (1.23, 1.34) |
| Pre-eclampsia | 7,729 (1.3) | 2,474 (1.7) | 1.29 (1.23, 1.35) |
| Eclampsia | 377 (0.1) | 113 (0.1) | 1.20 (0.97, 1.48) |
| **Other hypertensive disorders of pregnancy** | 11,711 (2.0) | 3,670 (2.5) | 1.26 (1.21, 1.31) |
| **Small for gestational age** | 68,333 (11.8) | 17,367 (12.0) | 1.02 (1.00, 1.04) |
| **Gestational diabetes** | 71,340 (12.3) | 18,842 (13.0) | 1.07 (1.05, 1.08) |

Abbreviations: ASCVD, atherosclerotic cardiovascular diseases; CI, confidence interval.

**Supplementary Table S4. Neonatal adverse events of offspring according to the presence or absence of maternal pre-existing atherosclerotic cardiovascular disease in full cohort (N= 5461222)**

| **Neonatal/infant outcomes** | **Number of events (%)** | | **Odds ratio***  **(95% CI)** |
| --- | --- | --- | --- |
|  | **Without pre-existing ASCVD** | **Pre-existing ASCVD** |  |
| **Major congenital malformations** | 189022 (3.6) | 6163 (4.2) | 1.09 (1.06-1.12) |
| **Neonatal composite morbidities** | 256876 (4.8) | 8174 (5.6) | 1.13 (1.11-1.16) |
| **ICU admission** | 245465 (4.6) | 8991 (6.2) | 1.19 (1.17-1.22) |
| **Seizures** | 129694 (2.4) | 3571 (2.5) | 1.11 (1.07-1.15) |
| **Neonatal death** |  |  |  |
| Within 1 month | 2052 (0.04) | 58 (0.04) | 0.92 (0.71-1.20) |
| Within 1 year | 8434 (0.2) | 243 (0.2) | 1.03 (0.91-1.17) |
| **Long-term outcomes** | **Number of events**  **(Cumulative incidence%)** | | **Hazard ratio***  **(95% CI)** |
|  | **Without pre-existing ASCVD** | **Pre-existing ASCVD** |  |
| **Neurodevelopmental disorders** | 803325 (18.9) | 21395 (21.1) | 1.09 (1.07-1.10) |
| **Any cause of death** | 14108 (0.3) | 359 (0.3) | 0.97 (0.87-1.08) |

Abbreviations: ASCVD, atherosclerotic cardiovascular diseases; CI, confidence interval; ICU, intensive care unit.

*Adjusted for birth date, income, residential area, maternal age at delivery, number of birth prior current births, maternal history of stillbirth, and comorbidities before pregnancy (hypertension, diabetes mellitus, hyperlipidemia and malignancy).

**Supplementary Table S5. Sensitivity analysis for Neonatal adverse events of offspring according to the presence or absence of maternal pre-existing atherosclerotic cardiovascular disease among only first-time mothers (N=411159)**

| **Neonatal/infant outcomes** | **Number of events (%)** | | **Odds ratio**  **(95% CI)** |
| --- | --- | --- | --- |
|  | **Without pre-existing ASCVD** | **Pre-existing ASCVD** |  |
| **Major congenital malformations** | 12979 (3.9) | 3362 (4.2) | 1.07 (1.03-1.11) |
| **Neonatal composite morbidities** | 15716 (4.8) | 4259 (5.3) | 1.11 (1.08-1.15) |
| **ICU admission** | 16701 (5.1) | 4705 (5.8) | 1.16 (1.12-1.20) |
| **Seizures** | 8098 (2.5) | 2175 (2.7) | 1.10 (1.05-1.16) |
| **Neonatal death** |  |  |  |
| Within 1 month | 110 (0.03) | 28 (0.03) | 1.04 (0.69-1.58) |
| Within 1 year | 440 (0.1) | 128 (0.2) | 1.19 (0.98-1.45) |
| **Long-term outcomes** | **Number of events**  **(Cumulative incidence%)** | | **Hazard ratio**  **(95% CI)** |
|  | **Without pre-existing ASCVD** | **Pre-existing ASCVD** |  |
| **Neurodevelopmental disorders** | 52154 (21.5) | 12756 (23.4) | 1.08 (1.06-1.10) |
| **Any cause of death** | 740 (0.3) | 193 (0.3) | 1.07 (0.91-1.25) |

Abbreviations: ASCVD, atherosclerotic cardiovascular diseases; CI, confidence interval; ICU, intensive care unit.

**Supplementary Table S6. Sensitivity analysis for Neonatal adverse events of offspring according to the presence or absence of maternal pre-existing atherosclerotic cardiovascular disease among only participated in health examination (N=****313024)**

| **Neonatal/infant outcomes** | **Number of events (%)** | | **Odds ratio***  **(95% CI)** |
| --- | --- | --- | --- |
|  | **Without pre-existing ASCVD** | **Pre-existing ASCVD** |  |
| **Major congenital malformations** | 10689 (4.2) | 2647 (4.3) | 1.03 (0.98-1.07) |
| **Neonatal composite morbidities** | 12778 (5.1) | 3362 (5.5) | 1.09 (1.04-1.13) |
| **ICU admission** | 13638 (5.4) | 3767 (6.2) | 1.15 (1.10-1.19) |
| **Seizures** | 5399 (2.1) | 1377 (2.3) | 1.05 (1.00-1.11) |
| **Neonatal death** |  |  |  |
| Within 1 month | 106 (0.04) | 22 (0.04) | 0.85 (0.54-1.35) |
| Within 1 year | 311 (0.1) | 83 (0.1) | 1.09 (0.86-1.39) |
| **Long-term outcomes** | **Number of events**  **(Cumulative incidence%)** | | **Hazard ratio***  **(95% CI)** |
|  | **Without pre-existing ASCVD** | **Pre-existing ASCVD** |  |
| **Neurodevelopmental disorders** | 32693 (20.4) | 8455 (21.8) | **1.07 (1.04-1.09)** |
| **Any cause of death** | 473 (0.3) | 120 (0.3) | 1.03 (0.85-1.26) |

Abbreviations: ASCVD, atherosclerotic cardiovascular diseases; CI, confidence interval; ICU, intensive care unit.

*Adjusted for birth date, income, residential area, maternal age at delivery, number of birth prior current births, maternal history of stillbirth, comorbidities before pregnancy (hypertension, diabetes mellitus, hyperlipidemia and malignancy), body mass index, and smoking status.

**Supplementary Table S7. Competing risk analysis of neurodevelopmental disorders in offspring by maternal pre-existing atherosclerotic cardiovascular disease status**

| **Long-term neurodevelopmental disorders** | **Sub hazard ratio**  **(95% CI)** |
| --- | --- |
| **Overall** | 1.08 (1.07-1.10) |
| **Without APO** | 1.08 (1.06-1.10) |
| **With APO** | 1.09 (1.07-1.13) |

Abbreviations: APOs, adverse pregnancy outcomes; ASCVD, atherosclerotic cardiovascular diseases; CI, confidence interval.

Subdistribution hazard ratios (sHRs) with 95% confidence intervals (CIs) estimated using Fine–Gray competing risk models. Death was considered a competing event.

**
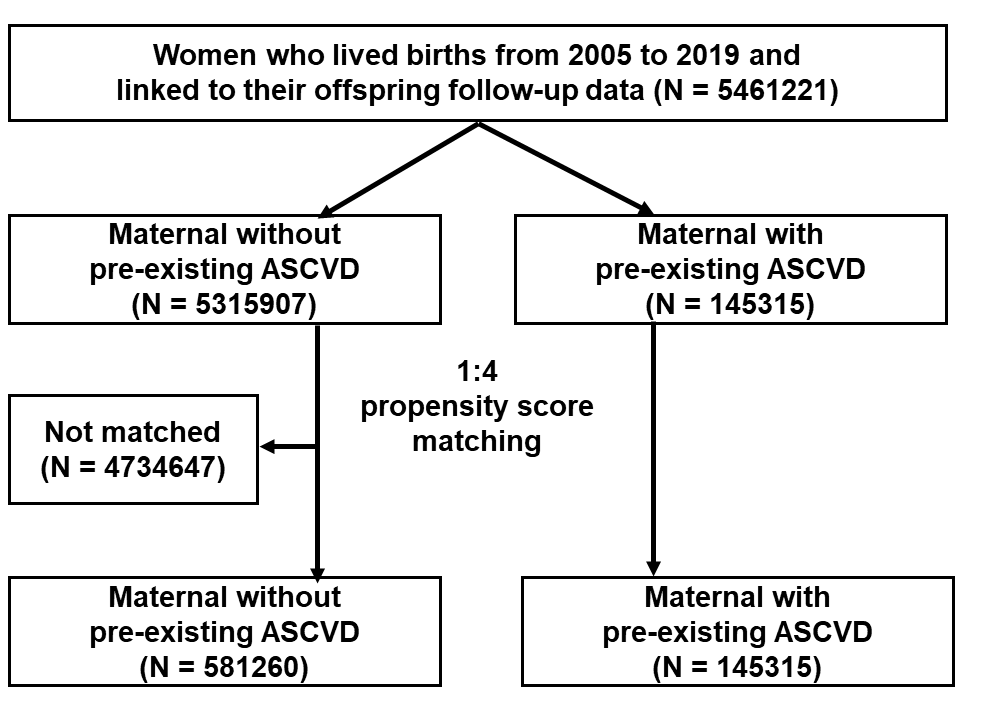
**

**Supplementary Figure S1. Study flowchart**

Abbreviations: ASCVD, atherosclerotic cardiovascular diseases.
